# Parent-carer experiences using a peer support network: a qualitative study

**DOI:** 10.1186/s12889-023-16666-9

**Published:** 2023-10-16

**Authors:** Rebecca Gudka, Charlotte Kelman, Eleanor Bryant, Bushra Farooq, Vashti Berry, Gretchen Bjornstad, Faith Martin, Sarah-Lou Glover, Abigail Russell

**Affiliations:** 1 University of Exeter Medical School, University of Exeter, Exeter, UK; 2 University of Bristol, Bristol, UK; 3 Children and Young People’s Mental Health Research Collaboration, University of Exeter, Exeter, UK; 4 Coventry University, Coventry, UK; 5 Parental Minds, Honiton, UK

**Keywords:** Holistic care, Wellbeing, Behaviour change intervention, Peer support, Parents, Carers, Qualitative, Mechanisms

## Abstract

**Introduction:**

Parent-carers of children and young people (CYP) with mental health problems are at greater risk of poor outcomes, such as poor physical and mental health. Peer interventions for parent-carers of CYP with disabilities may improve parent-carer outcomes. This qualitative study investigates parent-carer experiences of using Parental Minds (PM), a multi-component peer support service for parent-carers of CYP with disabilities.

**Methods:**

Twelve current service-users and four staff/volunteers at PM participated in one-to-one semi-structured interviews. All participants were white females, except for one service-user who was male. All interviews were recorded and transcribed verbatim. Thematic analysis of results was used to explore perceived benefits and disadvantages of PM and possible behaviour change mechanisms.

**Results:**

Three themes and eight subthemes were identified. Participants identified that internal and external factors influence their self-concept. The identification of themselves as a priority, and empowerment by reassurance and affirmation lead to improved parent-carer self-efficacy and agency to better care for their CYP. Participants described the difficulty of speaking honestly with friends and family about what they experience because it is perceived as different to what “normal” parents experience. From participant accounts, PM enables the construction of a support network and links external services to help manage family circumstances rather than offer curative treatment/intervention. Proactive and immediate advice which is constantly and consistently available was valued by participants. Participants expressed the need for a flexible range of service components which provide holistic support that encompasses both health and social care.

**Conclusions:**

PM was perceived to be beneficial as a multi-component peer support service which increases parenting self-efficacy and empowerment, reduces isolation, improves access to services, and is tailored to individual needs. Parent-carers reported benefits in parenting and wellbeing practices. The development of a refined logic model will inform a future study of the effectiveness of PM on parent-carer outcomes.

**Supplementary Information:**

The online version contains supplementary material available at 10.1186/s12889-023-16666-9.

## Introduction

The prevalence of mental health problems and special educational needs in children and young people (CYP) is rising in the UK [1, 2]. The Office of National Statistics (ONS) reported intentional self-harm as a leading cause of death for CYP aged 5–19 [3]. Compared to one in nine 5–16 year olds in 2017, one in six 5–16 year olds were found to have a probable mental health condition in 2020 [1]. Mental health conditions can be defined as disabilities if they impair everyday functioning and have a persistent effect over 12 months [4]. Other estimates suggest that two in five CYP score above the clinically relevant thresholds for emotional problems, conduct problems or hyperactivity [5]. This means that a huge number of parents are acting as parent-carers for their children, managing their children’s mental health and emotional needs.

CYP with mental health disorders are less likely to develop additional depression and panic disorders after seeking contact with mental health services in the UK [6, 7]. However, CYP and their families face barriers to accessing support including lack of information about how to seek help, the perception that they will be disregarded by healthcare professionals (HCP), long waiting lists for support from mental health services, short appointment times which are not deemed long enough by parent-carers, and stigma associated with help seeking [8–11].

CYP are not autonomous, they are cared for by parents and guardians who form key figures in their immediate environment and development [12]. Parents and guardians caring for CYP with disabilities, including mental health problems, are at increased risk of household disruption and overall lower family functioning; loss of work or employment opportunities leading to financial disadvantage; suffering from stress, anxiety, feelings of guilt and in some cases the onset or worsening of clinical depression, and poorer physical health [1, 13–18]. Parents also report impacts on siblings of CYP who self-harm, such as feelings of stress and additional responsibility [18]. Additionally, parents of CYP with mental health problems have described feeling isolated as a result of a lack of understanding or empathy from teachers, HCPs, family and friends, and felt they would be labelled a bad parent should they seek support [10, 19]. Eaton et al. demonstrated the effects of external stigma on mothers of CYP with mental health difficulties, including social avoidance and self-doubt, which can become internalised as shame and poor parenting self-efficacy [14]. Studies have shown that service needs of parents and families of CYP with disabilities are complex and multi-faceted. They include better information provision, socio-economic support which enhances self-efficacy, support to extend social-networks, practical support to offer parent-carers a break from the responsibilities associated with caring for a CYP, and recognition of their role as a caregiver [20–22].

Bi-directional and reciprocal relationships between parent and CYP mental health suggests that improvements in the mental health of one individual may confer benefits for family members [12, 23]. Furthermore, family outcomes may be improved by parent-targeted interventions, but there are a lack of programmes to provide such support, demonstrated by recent reviews [24, 25]. Peer support interventions can be defined as “non-hierarchical interpersonal processes promoting mutual healing in the context of community, characterized by equitable relationships among people with shared experiences” [26]. There is unclear evidence about whether peer support interventions improve psychological or psychosocial outcomes for parents, and generally the studies are small scale and lack elicitation of mechanisms of behaviour change [27, 28]. Qualitative findings suggest that peer support networks act as a means of providing shared social identity, practical advice and resources, and personal growth, which in turn may increase parent-carers' wellbeing and their perceived ability to care, and have been shown to be highly valued by parent-carers [19, 27, 29–31]. Additionally, Public Health England emphasised the need for community-based intervention to improve health and wellbeing [32, 33]. There is a particular need for additional support for parent-carers in South-West England, having the second highest rate of child and adolescent mental health disorders across English regions (15.5%) [34].

### Parental Minds

**Fig. 1:**
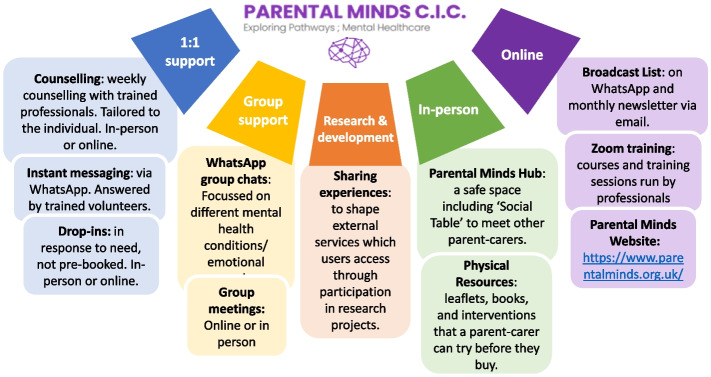
A visual representation of support components provided by Parental Minds Parental Minds offer a range of support options; service-users can create a bespoke service by using any combination of the components provided. Support is delivered in a variety of formats, service-users can choose to take part in one-to-one or group support, both of which can be delivered online or in person. Service-users are also given regular opportunities to share their experiences with researchers to inform and help develop external services

Parental Minds (PM) is a service for parents or any adult carers of CYP living with a mental or physical health issue or disability. Parent-carers self-identify if they are a parent or carer, and feel that they need support relating to a CYP who has any form of mental, neurodevelopmental or physically impairing condition that impacts on the CYP’s mental health. There are no restrictions on the age of the CYP involved or the level or chronicity of their need. This could range from, for example, an adoptive parent of a 5-year-old child exhibiting school refusal, to a family friend who supports a 19 year old with intellectual and physical disability. The most common needs of CYP that PM users report include neurodevelopmental disorders, emotional dysregulation and self-harm, and challenges with school attendance. PM is based in the East-Devon area [35]. We consider the overarching description of PM to be peer support, as it is led and staffed by adults who are themselves parent-carers with lived experience of supporting a CYP with mental health or neurodevelopmental challenges, with the minority of what is offered involving trained professionals. This fits with others’ definitions of peer-support in which assistance is provided by a person who “possesses experiential knowledge of a specific behaviour or stressor and similar characteristics of the target population” [26, 36]. PM aims to provide support to help enable caregivers to better deal with the emotions, stress and difficulties of living with CYP with a variety of different mental health problems. PM offers interventions that fall under the MRC definition of complex and multicomponent [37]; including WhatsApp groups with parent-carer peers, in-person and online “Hub” sessions for parent-carers to meet and talk to peers, as well as one-to-one counselling from trained HCPs, and “try before you buy” intervention sessions for parent-carers to explore different therapies at low cost. The components offered by PM are summarised in Fig. 1. For further information about the organisation, funding, staffing and other detail about their operation, please see https://www.parentalminds.org.uk/.

### How do interventions work and improve experiences?

One common method used to understand the mechanisms through which psychosocial interventions such as PM may impact on the lives of those who engage with the service is to ascertain what changes are perceived by individuals, and how these changes come about – what aspects of their behaviour have changed. Prior to conducting a quantitative evaluation to ascertain if PM is “effective” at improving the lives of parent-carers and CYP, we first need to understand what these mechanisms of change may be, and therefore what would be relevant to measure. Behaviour change theory [38] advises building a ‘logic model’ showing theoretical pathways of behaviour change through components of an intervention, in order to understand the mechanisms through which outcomes are improved. As a starting point, qualitative research is an excellent vehicle for exploring the experiences of individuals to assess and refine a draft logic model, and to ascertain what the outcomes may be for individuals using a behaviour change intervention (BCI). BCIs are critical to improving health outcomes [39]. According to Social Cognitive Theory, peer-support interventions like PM may lead to improved health outcomes by changing the external environment and personal motivational factors through bespoke social support [40]. Furthermore, the Theory of Planned Behaviour [41] suggests that through peer and social components of the service, PM may be changing subjective norms, attitudes towards behaviour and perceived behavioural control. This in turn might lead to changing the intention to perform a behaviour and therefore change behaviour. We consider that PM and similar models of formalised peer support may be an effective intervention for supporting parent-carer mental health. Before testing effectiveness and prior to conducting this study, it is important to understand how and why PM might be effective.

### Aims and research questions

The aim of the current study was to understand how and why PM may be an effective service to support parent-carers and their wellbeing, and understand any potential detrimental impacts of PM. To achieve this, we addressed the following research questions:

1. What are the experiences of parent-carers of CYP with mental health problems using Parental Minds?
2. What are parent-carers’ perceived improvements in relation to health and wellbeing of themselves and their CYP after using Parental Minds? Are there any reported challenges?
3. What are the behaviour change mechanisms through which involvement with Parental Minds may improve outcomes for parent-carers?

## Methods

A qualitative research design was used, because qualitative research is appropriate for exploring and understanding experiences and the context behind them [42]. Qualitative findings cannot be generalised across populations and are not suitable to draw robust comparisons between individuals, but are valuable for insight into potential mechanisms of effect. Data were collected using semi-structured interviews, which are useful for encouraging participants to talk freely about their experiences, allowing a rich and detailed exploration of varied concepts within a pre-defined framework of topics [42]. The initial logic model of behaviour change that we developed for PM (see Additional file 1) was developed prior to interviewing participants based on a taxonomy of behaviour change methods [43] and consulting with a current service user at PM; this was then tested and refined through the qualitative interviews. A barrier to implementing and evaluating BCIs is the lack of standardised language to describe active components, therefore we used the Behaviour Change Taxonomy laid out by Michie et al*.* to develop the logic model [43]*.*

### Participants

The eligible population were individuals with lived experience as a parent-carer who were involved with PM as either staff or service user. Many of the staff at PM work for the organisation on a voluntary basis, and originated as service users, and so there is not always clear distinction between roles. Staff were defined as those who took on additional roles or had specialised training. At the time of writing, the majority of staff members at PM were volunteers, however for the clarity of the reader all paid and unpaid staff members will henceforth be referred to as “staff” to distinguish from PM service users. Participants were 12 PM current service users, three staff members who helped deliver PM services, and the founding director of PM, recruited using opportunistic sampling methods. To be included in the study, eligible service users were mental health caregivers (18 + years) to a family member, friend or colleague (of any age). Participants were required to be using any of the services offered by PM. All staff at PM have experience of supporting a CYP with mental health or neurodevelopmental challenges, and many have previously used PM services, and so staff were required not to be receiving any services from PM at the time of data collection.

### Procedure

Service users and PM staff were recruited following an invitation via the PM mailing list, which is sent to all current PM service users. At the time of data collection, there were approximately 150 PM service users. The invitation included an information poster with an expression of interest form. Individuals who filled out the form were followed up with detailed information leaflets and a phone-call from RG who offered the opportunity for participants to ask any questions regarding the conduct of the study: this step was important in ensuring that participants were aware that their participation and raw non-aggregated data would not be reviewed by the CEO, who has collaborated on the research study. After the first eight interviews had been conducted, participants’ demographic information was reviewed, and targeted recruitment took place to find the subsequent 10 participants with the aim of achieving maximum demographic variation within the target population.

Interviews were chosen rather than focus groups, in order to enable participants to share thoughts and experiences freely and to ensure there would be no impact on their potential interactions with other participants through their usual involvement in PM (e.g., ensuring service users were not identified to one another if they usually participated anonymously, or chose not to share details of their CYP’s difficulties widely within their PM peer interactions). These took place by phone-call or Zoom at a time convenient for the participant between mid-February and early March 2022. Interviews were conducted by RG, EB, CK and BF: two undergraduate research assistants and two PhD students respectively and had all undertaken qualitative methods training. None of the researchers had a prior relationship with any participants interviewed by them. SLG (the CEO of PM) supported recruitment by sharing study information and was interviewed for the study in their capacity as PM staff member, but was blinded to which service users and staff participated in the study. The research team who conducted data collection and analysis were independent of the PM organisation.

Participants provided written informed consent prior to their interviews, they were advised of their right to withdraw at any time or decline to answer any question they were uncomfortable with, and that the aim of the study was to find out about the use and their perceptions of PM services. All interviews were audio-recorded and transcribed verbatim. Transcriptions were checked by RG prior to analysis. Interviews lasted between 30*–*75 min.

Demographic information was collected from each participant at the start of their interview. Semi-structured topic guides were used to ask participants about the use and delivery of PM services and their perceptions of the benefits or challenges caused by using PM (see Additional file 2). Topic guides were initially developed using the logic model to inform questions and were reviewed and refined after each interview. Topic guides contained 12 questions, with prompts to help participants fully elaborate on their answers. The introduction of new topics by participants was encouraged, but topic guides ensured that answers remained on track. Interviewers were debriefed by AR after each interview to address any safeguarding concerns.

Ethical approval was obtained from the University of Exeter College of Medicine and Health (UoE CMH) Ethics Committee (Reference: 491982). This study was unfunded and is a collaboration between the Children and Young People’s Mental Health Research Collaboration (ChYMe), UoE CMH, and Parental Minds. A collaboration agreement between Parental Minds and UoE CMH was agreed and signed.

### Data analysis

NVivo version 11 was used to support qualitative analysis of the data, using Thematic Analysis as described by Braun and Clarke [44]. Thematic Analysis is a flexible qualitative analysis method which can be used to identify patterns within data, which assumes no theoretical position [44]. A coding framework was developed by RG and AR who read two transcripts and discussed summaries of each line of data, assigning key words to every line which were then consolidated into the initial list of codes. The codes were sorted into three deductive categories: services and need, mechanisms of change, and effects of change. This allowed the data to be organised by context during the initial coding stages. RG read and reread each transcript to familiarise herself with the data. RG and AR then independently coded each transcript using a constant comparison approach, which allowed codes within the existing framework to be merged, and new codes to be added as analysis continued. Clear exceptions (to the consensus of parent-carer experiences) and negative experiences were searched for and highlighted by RG. Two researchers coded the data to increase the inter-rater reliability of the analysis. Reliability across coders was checked and the percentage agreement across the two coders was 98%.

The coded data were summarised by RG and AR, with the summary of each code being discussed between the two researchers to identify a tentative list of subthemes. These subthemes were presented to the authorship team [excluding SLG] and discussions resulted in an early list of potential themes. The entire dataset was reread and recoded into the final subthemes to ensure they were credible in the context of the whole dataset and to ensure that data were coded in their most appropriate subtheme with no significant overlap. Subtheme titles and summaries were then written on post-its and physically arranged, a final structure of themes and subthemes was then established by RG.

## Results

All participants were white and over the age of 30, and all participants except P22 had been using PM for over 6 months. Table 1 provides a summary of participant gender, the gender of their CYP and their carer and employment statuses. The Thematic Analysis identified three themes relevant to research questions 1 and 2, with no clear distinction between themes aligning to each research question. Table 2 summarises the key themes and findings. We refer to “participants” to indicate the views only of PM service users who were interviewed. It has been specified where views of staff members are included, referring to them as “staff”.

**Table 1:** Characteristics of all study participants

Participant	Gender	Gender of CYP	Relationship to CYP	Self-perceived carer status	Employment status
P18	Female	Female	Parent	Part-time	Full-time
P20	Female	Female	Parent	Full-time	Part-time
P21	Female	Female	Parent	Part-time	Part-time
P22	Female	Female	Parent	Part-time	Part-time
P23	Female	Female	Parent	Full-time	Full-time
P25	Female	Male + female	Parent	Does not consider self as care-giver	Full-time
P26	Female	Male	Parent	Full-time	Other
P27	Female	Female	Parent	Does not consider self as care-giver	Full-time
P32	Female	Female *multiple*	Parent	Full-time	Self-employed
P33	Female	Female	Parent	Part-time	Full-time
P34	Female	Male	Parent	Part-time	Part-time
P35	Male	Male	Parent	Full-time	Full-time
SM28^a^	Female	-	-	-	Retired
SM29 ^a^	Female	-	-	-	Part-time
SM30 ^a^	Female	-	-	-	Self-employed
SM36^b^	Female	-	-	-	Full-time

^a^Staff members at Parental Minds were not asked about gender of CYP, relationship to CYP or self-perceived carer status because it was not deemed relevant to the study as volunteers/staff members who were also using Parental Minds services were excluded from the study with the aim of capturing a distinction between views of volunteers/staff and service users

^b^SM36 is the Founding Director of PM and has provided consent to be referred to as such in this publication

**Table 2:** Themes and summary of results

Theme	Subtheme	Description of theme and definition of terminology
**Parent-carer self-concept**	Identity and empowerment lead to agency	Description: Parent-carer self-concept is informed by internal and external factors, including parent-carers’ **identity** and **agency** as carers, and the **stigma** they experience. Participants acknowledge that PM helps them reframe their self-concept through **empowerment** Definitions: Identity is defined as an individual’s sense of self defined by (a) a set of physical, psychological, and interpersonal characteristics that is not wholly shared with any other person and (b) a range of affiliations (e.g., ethnicity) and social roles [45]. Agency is defined as the state of being active, usually in the service of a goal, or of having the power and capability to produce an effect or exert influence [46]. Empowerment is defined as the promotion of the skills, knowledge, and confidence necessary to take greater control of one’s life. In psychotherapy, the process involves helping clients become more active in meeting their needs and fulfilling their desires and aims to provide them with a sense of achievement and realization of their own abilities and ambitions [47]
	Transparency in a destigmatising environment	
**Not about a fix**	Proactivity	Description: From participants accounts, PM is a service which helps them to build a bespoke support network to manage their circumstances rather than ‘fix’ them by providing **proactive** advice and contacts, a non-judgemental environment built through **shared experience**, and a round-the-clock **safety net** for parent-carers Definitions: We define proactivity as preventative advice and behaviours which aim to help parents manage future situations, rather than reactive support for events which have already happened
	Shared experience	
	Safety net	
**Individual need**	Range of service	Description: PM is perceived as a unique service because of the **range of service** it offers, which meet each parent-carers’ individual needs. Service components encompass a variety of mental health difficulties and target many skills parent-carers wish to improve, the **flexibility** which accounts for **fluctuation** in wellbeing and use of services, and the understanding that **complex needs** call for **holistic support** Definitions: We define complex needs as two or more needs which affect a person’s wellbeing and are unique to that person and their circumstance. Holistic health is defined as a concept that medical practice, in the prevention and treatment of disease, should focus on the whole person—including physical, mental, spiritual, social, and environmental aspects—rather than on disease symptoms alone. Major features of holistic health include patient education about behavioural and attitudinal changes that promote and maintain good health and well-being, and patient self-help and participation in the healing process through diet, exercise, and other measures [48]. In the context of this paper, we refer to the non-clinical aspects of holistic health
	Fluctuation and flexibility	
	Complex problems, holistic solutions	

### Parent-carer self-concept

#### Identity and empowerment lead to agency

Support services for families with CYP who have complex needs were described as child-centric; participants felt their viewpoints and support needs were excluded by HCPs. Therefore, most participants identified the need for specific support for themselves as separate to the support received by their CYP.

*“[PM] was about the parent. When you've got somebody who you're supporting there is so much emphasis on what support they can get... I was struggling as much as she was and I just needed somebody to just talk to me and to listen to me” (P20)*

Most participants said that PM helped them to understand that identifying themselves as a priority was necessary to best support their CYP: “*[PM] taught me that, that to help your children, you need to be in a good place yourself” (P25).* In addition, participants felt less burden as carers when they understood that they were not the sole agent of change in their CYP’s life.

*“The biggest thing that I've taken on board from PM is… acknowledging that and realising that actually it is [my child’s] journey, it just takes that pressure off of me a little bit” (P20)*

Participants talked about feeling empowered by the reassurance and affirmation that they are “*on the right track” (P21)* which improved self-efficacy, leading to them being able to better support their CYP.

*“That reassurance and just that support to keep doing the things you are doing… in turn then obviously hopefully have the benefit to my daughter in that she will have better side of me because I feel I'm more able to help” (P22)*

Through the interventions offered from PM, participants felt they could *give back* to others in the group from a “*position to support people”* (P26). Participants’ identities within the group were described as “*merg[ing] between me being offering support and me needing support”* (P21); both roles were viewed as beneficial by participants.

#### Transparency in a destigmatising environment

Some participants identified their experiences as parent-carers as “*outside of the normal realm of people's experiences” (P21)*, with “*few and far” (P22)* people sharing similar experiences. Participants mentioned feeling they were a burden on people who did not share their experiences was one reason why they could not speak “*honestly to anybody at home or family” (P33)*. Thus, access to a trusted, purpose-built, “*safe space”* (P22) at PM meetings was considered important.

*“You don’t want to be always talking to your friends about your problems, you just want to have a normal relationship with your friends, and the same with family, so it’s nice to have a group of people that you can just talk about stuff you’re worried about… you don’t feel like you’re placing a burden on anyone” (P22)*

Some participants felt that stigma around receiving mental health support prevented them from being open about using PM, especially for fear of their CYP’s peers finding out and the impact this may have on their CYP.

*“It’s been tricky because it’s my [child's] private life and [they don't] want everyone to know what’s going on in her world… I haven’t told everybody because some of them have got kids, peers their age” (P22)*

Participants who had active roles in the community felt it was inappropriate to reveal their identity to the group if they knew other service users because it would harm their reputation, or make them less trustworthy in their jobs, so felt that they could not participate fully in group sessions.

*“We’re very well known in the community, that can make it a bit awkward in that I don’t necessarily want my private life out there or my children’s lives out there when there are people in the group who may be parents of children that I work with” (P25)*

### Not about a fix

#### Proactivity

Several participants discussed the practical, preventative advice that PM offers, which helped them to avoid reaching crisis point with their CYP. For example, one participant mentioned *practical “How to Talk guides”* (P22), and another received advice about devices her child can use to charge their phone while out because running out of battery caused them to have “*meltdowns”* (P26). This was described by one participant as signposting to training and resources “*from a place of hope” (P21).* Service users and staff liked the delivery of information on WhatsApp because “*it's easily accessible, short and easy to read” (P27).* Several participants discussed the benefits of being put in touch with specialists from different services by PM. Participants felt able to approach services that would normally be inaccessible and were given specific instructions about how to apply for services, which improved their understanding of the help-seeking process.

*“Someone was invited from DIAS [Devon information and support] … They bring in people that you can then talk to, which normally would be much more difficult and would be more barriers. In that sense, that information is very useful and it’s also very person-specific then because you can ask questions that are relevant” (P32)*

#### Shared experience

Most participants felt that the PM Hub and WhatsApp groups were safe and non-judgemental environments which were built on the knowledge that all members of the group, including staff, share experiences with each other. Participants believed that they would receive more empathy from those who have “*actually lived through what is happening” (P23)* to them.

*“It’s nice to know that… there are people that I can reach out to that will not pass any judgement, but will understand because they’ve probably gone through something similar” (P22)*

Through the Hub meetings and WhatsApp groups, participants received positive affirmations, self-comparison of their approaches with those used by other parent-carers, and learned from other parent-carers’ experiences, all of which were deemed useful for improving their ability to look after themselves and their CYP. Most participants agreed that they put “*extra trust and confidence through the [services] that have lived experience” (P18)* compared to websites and NHS services.

Some participants found that hearing other people’s experiences of their CYP’s difficulties, particularly those involving self-harm, was “*scary or upsetting”* (P18) and caused distress due to concerns that their CYP might progress to similar levels of distress. Furthermore, one participant described it as “*overwhelming”* and “*distressing [to know] that there are so many people who feel let down by the [NHS]*” (P21).

#### Safety net

One staff member described how “*the WhatsApp groups are really interactive, and people can access them nearly any time” (SM29).* Most participants made concurring remarks that PM can be used as a safety net or “*security blanket… that you can access as much as you want to” (P20).* Some participants who felt unable to reach out for help found the check-in calls and messages useful, one participant described it by saying “*there’s always someone who shows that they care, and they’re thinking in the background about me” (P32)*.

Participants described the limitations of other services, for example one-off appointments or a limited number of sessions, whereas PM did not discharge parent-carers if they did not access the service, and continued involvement was encouraged through allowing parent-carers to transition to a more supportive role, which is described as beneficial by both service users and staff.

*“A lot of services they tend to get rid of you after a bit, but that’s different with PM, you can come back and use them as and when you need to” (P26)*

*“At Young Minds you can sign up and have a chat as a one-off chat and actually I needed… somebody who knows a bit about the history so you don’t have to explain each time” (P22)*

### Individual need

#### Range of service

Staff observed that PM saved people *time and anxiety* by providing a “*wide range of different resources… so people don’t have to skip around looking for stuff that’s good quality” (SM29).* Most participants perceived the varied support as a unique benefit of PM because* “it can potentially help across a range of issues” (P22).*

*“It's really good that PM have set up lots of different groups that you can access… I think it's really good to be able to be sign posted to different groups to get that support. It's just invaluable” (P20)*

However, two participants mentioned that PM being a “*jack of all trades*” and “*covering too much ground” (P32)* concerned them because they worried that attention to specific difficulties would be overlooked by the growing service, and that they were becoming overwhelmed by the number of services PM had to offer: “*There’s been more to offer than I’ve been able to take on board” (P35).*

In general, when discussing the type of support available, interviewers observed that both service users and staff used imprecise language and their own set of terms. Furthermore, some participants were unaware of who they were talking to in WhatsApp groups, whether it was a staff member, councillor, or a helpful parent-carer. Sometimes this led to participants feeling apprehensive or nervous to contribute to WhatsApp group conversations.

#### Fluctuation and flexibility

Most participants described increasing the intensity of their use of PM when their mental health, or that of their CYP, was declining, as they perceived PM could supply immediate solutions to challenges they were facing.

*“I guess when we’re a bit unstable is when I’d use them most, because I haven’t got time when we’re at crisis point” (P23).*

Some participants talked about the difficulty in attending face-to-face sessions during these times because they could not leave their CYP alone or did not have the time or energy to attend face-to-face meetings. One participant said they did not attend the face-to-face sessions “*not because they don’t offer them*”, but because their CYP “*would be unsafe to leave”* (P23).

Due to the broad range of support for different issues, and ways in which support was delivered, many participants valued the ability to “*cherry pick” (P26)* information which was relevant to them and in the most usable format. Freedom of choice about how engaged participants could be with WhatsApp groups and online sessions was viewed as beneficial because “*you can either respond or you don’t… there’s no pressure” (P22).*

*“The Zoom [meetings] are quite good because you can be involved or not involved as you want. With the home-ed one I was fine to put video on, and we got quite chatty, but some things you might not be so willing to be open” (P26)*

#### Complex problems, holistic solutions

Participants discussed the difficulties of finding support for parent-carers, believing they did not “*fit the box” (P18)* for provisions already available through their GPs: “*there isn't the services out there for people like me” (P23).* Services for parents that were recommended by HCPs were considered too generic for the complex and heterogeneous support needs of each parent-carer, with what was described by one participant as “*specific frameworks of support”* (P26) for individual difficulties rather than all the parent’s needs.

*“Lots of professionals, sadly, rather than looking at the needs, … they’re not seeing the person and they’re not seeing a holistic view” (P21)*

Participants described the remit of support offered by PM as being holistic because it identified people’s individual needs and offered solutions which encompassed many aspects of their lives, including both health and social care.

*“Some services would be like “we deal with this, but we don’t deal with anything else” but with [PM] it’s very holistic… they don’t specifically deal with benefits, foodbanks or education healthcare plans and stuff, but they will put you in touch with somebody that does” (P26)*

## Discussion

Parent-carers perceived PM to be a service which helped them reframe their identity from the only agents of change in their CYPs lives to supportive agents. Parent-carers were helped to identify and prioritise their own needs which increased their belief in their own ability to support their CYP. Parent-carers also reported that empowerment and affirmation improved their ability to look after their CYP because it increased their parenting self-efficacy and confidence. These perceived benefits are not unique to PM, with previous research finding similar benefits of parent-to-parent support [29, 30, 49]. In addition, parent-carers perceived their ability to offer support to others as beneficial. This is consistent with previous research suggesting similar feelings of satisfaction and personal growth when parent-carers felt they could offer help to others [30, 50]. Identification of self as a role model (i.e., the perception that one’s behaviour may be exemplary to others) is an evidence-based behaviour change mechanism present in taxonomies, which in this case could be encouraging positive parenting practices [43].

In line with previous findings in this field, parent-carers highlighted the importance of having a purpose-built safe space to create a shared sense of belonging and community [19, 29, 30, 50]. The perception that they were different to “normal” parents and burdensome on others were reasons participants reported for not openly speaking to family and friends about the difficulties they were experiencing. Their experiences of PM were that it restructured and supplemented their social environment with parent-carers who were understanding and empathetic [43]. Studies have found that social support is protective, and reduces the feelings of isolation and stigma which can be self-imposed when parent-carers avoid social situations due to their CYPs disability [30, 31, 51]. Despite most parent-carers feeling that they could speak freely in PM group sessions, there were instances of service users feeling that they could not participate fully for fear of professional judgement in their jobs because they knew other service users in an external context. If demand is sufficiently large, there may be potential for PM to set up a separate WhatsApp group for those parent-carers working in local health and social care services, who may have conflicts of interest within the usual groups, to ensure all parent-carers feel they can safely access PM. Additional education or information resources for other parents to destigmatise help-seeking and raise awareness that everyone should have equal access to services, regardless of their profession or other characteristics, may also help to tackle the root of the problem, although this may not resolve the internalised stigma that some PM users experience.

Parent-carers described receiving practical advice from PM which enabled them to learn from other parent-carers and people with lived experience who have tested methods to help avoid crisis situations with their CYP. This is recommended in the literature, which shows that parents who have CYPs with disabilities need and value practical advice, but seldom receive it due to HCPs lack of awareness of parent-carers needs [31, 52]. One of the most frequent overall barriers related to accessing services is lack of information about where and how to seek help [8, 9]. Our findings show that PM provides parent-carers with direct contact and networking opportunities with professionals from services that parent-carers otherwise felt were inaccessible due to lack of information about how to access them. This mechanism of support has been shown to benefit parent-carers in previous research [53]. The role of PM goes beyond signposting parent-carers to services by also teaching them how to access and make best use of the relevant service.

While most parent-carers considered shared experiences as important because it increased empathy and decreased judgement received from others, some were distressed by the extent of other CYPs mental health conditions, particularly those involving intentional self-harm. This is concurrent with previous research where professionals expressed concern about the burden being placed on those listening to other parent-carers’ challenging experiences [54]. On the other hand, shared experience may improve parent-carers knowledge and parenting self-efficacy by providing opportunities for social comparison and affirmational/informational support from what parent-carers perceive as a credible source [43, 52, 54]; our findings highlight the need to ensure experiences are shared in a safe manner that does not cause distress. Parent-carers often continued their journey with PM by acting as a supportive member in the network or progressing onto being a volunteer/staff member. While this is described as beneficial by service users and staff members, previous research indicates that parents’ wellbeing may be compromised by hearing about issues they were still experiencing from other parents when taking on a supportive role [50]. Refinement of the service could include a structured and objective pathway for parent-carers to progress to “supporter” status to allow PM to monitor parent-carer wellbeing. This pathway may consist of skills training such as mental health first aid courses to ensure that PM provide parent-carers with the skills to become “supporters” and staff members.

Addressing the complexity and holistic management of participants’ lives was considered vital by most participants, which is consistent with a recent study in which the disjointed, multi-agency system was considered a barrier to adequate support, with a lack of communication between schools and care providers perceived as a way of HCPs deliberately gatekeeping services [8, 55]. Our findings support recent calls for family-centred, integrated models within services for CYP [25, 55]. Staff members and service users emphasised the importance of having many services accessible in one place, across a range of difficulties, as they perceived this reduced anxiety in service users. Future research may use measures of parent-carer anxiety and wellbeing to assess whether these perceptions are true. Participants also described the importance of having a flexible service which could be accessed at times convenient for them. This is considered a barrier to accessing care in the literature [8]. The ability to engage at a time convenient to them coupled with continuity of care (even when use is limited) means the parent-carer can access PM when they want to. Most describe their use of PM increasing when their mental health or that of their CYP was declining, but before crisis point was reached. Participants stated that face-to-face sessions were difficult to attend during challenging periods, so continuing to deliver blended services which include online sessions may be beneficial for these parent-carers.

To alleviate concerns from service users about who they are receiving advice from PM could ensure that all staff members are easily identifiable on WhatsApp and in the long term may consider creating a bespoke app or messaging service. This may reduce exposure to distressing content and encourage nervous parent-carers to feel they can contribute freely. There is no specific research on this area, though interestingly a study of a community-based programme for parent-carers found that groups of 6–12 were the optimum size for gaining varied perspectives and facilitation of conversations [56]. PM may also consider streamlining the language used to describe its interventions to avoid parent-carers feeling overwhelmed or confused by which services are available to them. Future studies should explore the optimum group size for peer support organisations, considering both online and face-to-face groups, and explore which support elements are most valued by parent-carers and most effective to enable similar organisations to focus their resources on services that are most likely to benefit their service users.

**Fig. 2:**
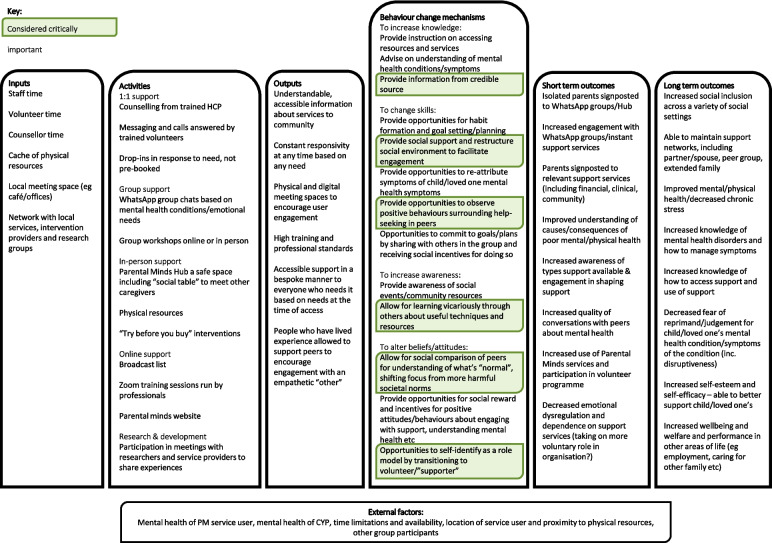
Refined logic model: representation of activities run by Parental Minds and their outputs Behaviour change mechanisms, short term outcomes and long-term outcomes were initially developed, then refined using the qualitative findings. Behaviour change mechanisms which were viewed as important by participants or abundant in the findings are highlighted in green

The findings from this study are largely consistent with the Logic Model. The refined logic model presented in Fig. 2 shows the behaviour change mechanisms that were identified as important or emphasised by the findings from this study. The outputs described in the logic model have largely been met according to the qualitative evidence received from participants, with the exception of high training and professional standards, which were not discussed by participants. Aside from this, the outputs mirror the themes and subthemes discussed in our findings. While some of the short-term and long-term outcomes have been discussed by participants, future research should use the Logic Model to develop aims and measures which empirically and quantitatively test the effectiveness of PM as an intervention for parent-carers of CYP with complex needs.

### Strengths and limitations

As previously mentioned, the evidence regarding whether peer support interventions improve parent-carer outcomes are generally limited by a lack of elicitation of behaviour change mechanisms. This study has strengths in that it explicitly explores potential mechanisms of behaviour change which may be occurring when parent-carers use PM. It uses a qualitative design to understand the theoretical pathways through which PM may be effective.

A significant strength of the conduct of this study is that it gathers evidence from parent-carers directly, when they often feel unable to participate in research due to access requirements. It was noted from several participants that they were only able to take part in the research because it was conducted remotely, at a time and place that was convenient and comfortable to them. Remote interviews mean that participants can take part from an environment which they are comfortable in, which may enable them to answer questions about difficult topics with more ease, but by using videoconferencing, they could still build rapport with the interviewer similarly to face-to-face interviews with use of verbal and non-verbal communication. The sample for our study was self-selected. Information about the study was circulated equally to all service users and staff, however those who chose to participate may have been more proactive and engaged with PM than those who chose not to.

The main limitation of this study is that it is lacking in diverse participant representation. As of 2020, the Southwest of England is the third least ethnically diverse area in England and Wales [57]. At the time of writing this paper, PM had very few male service-users and service-users of black and minority ethnic (BME) backgrounds. In addition, most female PM service users were over the age of 30, so there were few younger mums using PM. Whilst targeted messages to encourage participation were used, we were unable to recruit from BME backgrounds, and only one male was recruited. Reported perceptions of PM therefore may not be representative of fathers or parent-carers from diverse ethnic backgrounds, but they do represent the majority of current PM service-users. Previous research has shown that fathers’ help-seeking behaviour is influenced by their knowledge of depressive symptoms and stigma around masculine norms, and their partner and HCPs’ awareness of paternal mental health [58]. Therefore, we recommend that to engage more fathers in their services, PM could promote awareness to their female service-users and HCPs they are affiliated with. Male potential service-users may have fundamentally different needs that can only be met by a different service model. For example, models such as that of 'Dangerous Dads', who run outdoors activities and programmes for fathers and male carers of CYP of all ages, may be more appropriate, rather than assuming men will or should engage with the same service provision models as women. A recent systematic review found that local development of services to suit cultural environmental needs are important because there is no one-size-fits all approach to interventions for parent-carers [25]. Therefore, the findings from this paper may not be applicable to the wider population of parent-carers outside of the East-Devon area or to other parent-carer support groups but will help PM suit its service provision to the local community needs.

In this study we explicitly focused on the perspectives of parent-carers, and not the needs and views of the CYP that they care for two reasons. Firstly, the focus of this piece of research was on perceived changes for parent-carers to refine our logic model, and secondly, not all CYP of parent-carers knew about their parent-carers engagement with PM. Future research with samples of CYP, or with both parent-carers and CYP could add additional insight and give voice to CYPs own experiences and views.

A drawback to qualitative research is that results are not generalisable to all peer support networks in the wider population of the UK because of the focus on a particular population with unique characteristics [42]. The founder and CEO of the organisation was involved both throughout the research process and as a participant which increases the potential for bias in our findings. This is not uncommon in studies evaluating interventions where the ‘intervention developer’ is also a co-researcher and potentially participant due to their multiple roles that are relevant to the research process (e.g., [19]) As a team and with the ethics committee we discussed the nature of the CEO’s involvement and considered inclusion of their data and earned authorship to be acceptable. Future research could consider the relative pros and cons of working closely with intervention developers in the research process.

## Conclusion

In this qualitative analysis of a support service for parent-carers of CYP with mental health problems, using Thematic Analysis we generated three themes. Within the themes of *Parent-carer Self-Concept*, *Not About a Fix*, and *Individual Need*, our findings highlight the challenges faced by parent-carers and the need for individualised, multi-component peer support to help reduce stress and improve wellbeing and wellbeing practices. The majority of parent-carers who used PM perceived the service to be useful and beneficial. Service-users perceived PM to be a uniquely valuable service because it improves their self-efficacy and increases tangible support available to parent-carers by improving their access to other means of health and social care. Other services should invest in parent-carers and families, because this research shows the benefits of targeted parent-carer support. Nevertheless, some detriments to PM service use were discussed, and recommendations have been made, such as the implementation of a more objective pathway through “supported” to “supporter” and more regulated WhatsApp groups with easily identifiable facilitators. Some mechanisms of behaviour change which may be influencing parent-carer outcomes are identification of self as role model, restructuring the social environment, social support, and instruction on how to perform a behaviour. This study has developed a logic model which presents a theory of change that can be more rigorously tested in a future study of effectiveness of PM on parent-carer outcomes.

### Supplementary Information

**Additional file 1.** Initial logic model.

**Additional file 2.** Topic guides.

## Data Availability

The datasets generated and/or analysed during the current study are not publicly available to maintain the privacy of participants but are available from the corresponding author on reasonable request.

## Abbreviations

CYP: Children and young people
PM: Parental Minds
HCP: Healthcare professionals
