# Supplementary material for: Parent-carer experiences using a peer support network: a qualitative study
Source: BMC Public Health. 2023 Oct 16;23:2007. doi: 10.1186/s12889-023-16666-9 (PMC10577900; doi:10.1186/s12889-023-16666-9)
Supplement: Supplementary file 1 — Additional file 1. Initial logic model. [file 12889_2023_16666_MOESM1_ESM.pdf]

# Additional file 1: Initial logic model

The initial logic model of behaviour change, developed in consultation with a current Parental Minds service user, using Michie *et al.*'s taxonomy of behaviour change methods (43).

**Inputs**

Staff time

Volunteer time

Counsellor time

Cache of physical resources

Local meeting space (eg café/offices)

Network with local services, intervention providers and research groups

**Activities**

1:1 support
- 1:1 meetings with healthcare professional
- 1:1 support from 'listening ear'
- 1:1 messaging and calls answered by trained volunteers
- 1:1 drop-ins in response to need, not pre-booked

Group workshops
- Focussed on general behavioural strategies. 6-12 participants per group

In-person support
- Parental minds hub a safe space including "social table" to meet other caregivers
- Physical resources including leaflets, books and interventions that a caregiver can try before they buy

Online support
- Broadcast list on WhatsApp and monthly email newsletter
- Parental minds website

Research & development
- Participation in meetings with researchers and service providers to share experiences

**Behaviour change mechanisms**

To increase knowledge:
Provide instruction on accessing resources
Advise on understanding of mental health conditions/symptoms
Provide information from credible source about consequences of poor mental health to encourage help-seeking

To change skills:
Provide opportunities for habit formation and goal setting/planning in sessions with trained professional
Provide social support and restructure social environment to facilitate engagement
Provides opportunity to re-attribute symptoms of child/loved one mental health symptoms to improve self-esteem/low caregiver self-efficacy
Provides opportunity to observe positive behaviours surrounding help-seeking in peers
Opportunity to commit to goals/plans by sharing with others in the group + receiving social incentives for doing so

To increase awareness:
Provides awareness of social events/community resources

To alter beliefs/attitudes:
Allow for social comparison of peers for understanding of what's "normal", shifting focus from more harmful societal norms
Provide opportunities for social reward and incentives for positive attitudes and behaviours about engaging with support, understanding mental health etc
Opportunity for parents to become volunteers to help others which would help them self-identify as a role model improving self-esteem/self-efficacy
Allow for learning vicariously through others about the consequences of certain beliefs and attitudes

**Outputs**

No. HCPs + professionally trained volunteers deliver support

Amount of support offered via 1:1 calls

No. 1:1 drop-ins attended

No. group workshops run to completion

No. attendees to hub + social table

Amount of physical resources given out/administered

No. people with regular interaction with Whatsapp groups

No. items included on broadcast list

**Short term outcomes**

Isolated parents signposted to Whatsapp groups/parental minds hub

Increased engagement with support groups/instant support services

Parents are signposted to relevant support services (including financial, clinical, community)

Improved understanding of causes/consequences of poor mental/physical health

Increased awareness of types support available & engagement in shaping support

Increased quality of conversations with peers about mental health

Increased use of Parental Minds services and participation in volunteer programme

Decreased emotional dysregulation and dependence on support services (taking on more voluntary role in organisation?)

**Long term outcomes**

Increased social inclusion across a variety of social settings

Able to maintain support networks, including partner/spouse, peer group, extended family

Improved mental/physical health/decreased chronic stress

Increased knowledge of mental health disorders and how to manage symptoms

Increased knowledge of how to access support and use of support

Decreased fear of reprimand/judgement for child/loved one's mental health condition/symptoms of the condition (inc. disruptiveness)

Increased self-esteem and self-efficacy – able to better support child/loved one's

Increased wellbeing and welfare and performance in other areas of life (eg employment, caring for other family etc)
