# Supplementary material for: Parent-carer experiences using a peer support network: a qualitative study
Source: BMC Public Health. 2023 Oct 16;23:2007. doi: 10.1186/s12889-023-16666-9 (PMC10577900; doi:10.1186/s12889-023-16666-9)
Supplement: Supplementary file 2 — Additional file 2. Topic guides. [file 12889_2023_16666_MOESM2_ESM.pdf]

Additional file 2: Topic guides

The topic guides were used to inform semi-structured interviews with participants. While the introduction of new topics was encouraged, interviewers were asked to ensure that all topics in the guides were covered.

Staff and Volunteers topic guide

To start off can you tell us a bit about yourself and why you started working for/volunteering with Parental Minds?

And what is your role, how do you carry it out?

- Which services do you support in the delivery of? *Here is a diagram which summarises the services offered if you need a reminder (or look at the parental minds website with them https://www.parentalminds.org.uk/)*
- Are there any services that you don't help to support but that you have experience of?
- How do parents use the services that you help to deliver?
- Why do most parents seek support from Parental Minds? For themselves or for their child?

In your experience, how do the services which you help to deliver affect Parental Minds service users?

- *Break question up into each service (or particular people they have supported if hard for them to think by service) they deliver if it helps stay on track*
- Can you tell me how you think the services provided by Parental Minds affects service users:
    - Knowledge of how to support themselves/loved ones
    - Skills of how to support themselves/loved ones
    - Awareness of how to support themselves/loved ones
    - Beliefs/attitudes of how to support themselves/loved ones
- *If they mention signposting* Can you tell me a bit about some of the services you're frequently signposting people to?

Thinking about your personal experience working for Parental Minds; is there a service or component in particular that you think service users benefit from?

- Can you tell me more about how this service benefits service users and their loved ones/children's lives?
- And how it benefits their loved ones/children?
- Are there any downsides to it?

And is there one which you have delivered but that you don't think are useful/beneficial? If any services affect service users negatively, can you tell me how?

- Would you suggest any improvements to this service?
- Have you experienced or seen any negative outcomes relating to caregivers who use Parental Minds?
- Are there any upsides to it?

What guidance or support do you receive from Parental Minds so that you can do your role? How do you feel about this?

What is your overall opinion about Parental Minds? What are the good bits and the bad bits?

If you were in charge of Parental Minds, would you change or add anything?

- Could you tell me a bit more about that?

As researchers we're interested in collecting qualitative research like we're doing now in this interview, and quantitative data such as numbers and statistics. What should we be looking to measure in terms of numbers to show improvements due to parental minds?

<u>**In other words, what should we be looking at to show improvements?**</u>

- (<u>*hear their ideas first then suggest and get their thoughts on the following*</u>) Some ideas might include caregiver mental health, child mental health and the number of times someone needs to call or access crisis services or other emergency services

What is the best way for us to ask you or service users for this information?

- Eg. In person interviews, through WhatsApp/messaging, online surveys, being asked directly by researchers or by Parental Minds staff)

Is there anything else you'd like to say about Parental Minds that we haven't asked about?

Parent-carer topic guide

To start off can you tell us a bit about yourself and how and why you got involved with Parental Minds?

- Can you elaborate on why you felt Parental Minds was a suitable resource to help you with those things?

Which services have you accessed through Parental Minds? How often have you used each one? What tools and information have Parental Minds given you to support your child/cared-for?

- *If you need reminding here is an image which summarises the services offered (or look at website here https://www.parentalminds.org.uk/)*
- If you've used more than one service, could you rank them in order of most to least helpful? If not, what makes that a challenge?
- Can you tell me how using Parental Minds has affected your:
    - Knowledge
    - Skills
    - Awareness
    - Beliefs/attitudes

Thinking about your personal experience using Parental Minds; is there a service in particular that you feel you have benefitted from?

- Can you tell me more about how this service has benefitted you?
- And how do you think has it benefitted your loved one/child?

And is there one which you have tried but that you haven't found useful/beneficial? If any services have affected you negatively can you tell me how?

- And has it negatively affected your loved one/child? Can you tell me how?
- Would you suggest any improvements to this service which would have helped you?

Can you tell me about what has influenced how often you use any of the services you've mentioned previously?

- *Break down into specific services they've mentioned if they're stuck*
- For example, do you use any services more if your child/loved one is feeling better/worse, or any other situation you find yourself in

Can you tell me about how your ability to provide support for yourself has changed since you've started using Parental Minds? How has this come about? (if unsure what we mean: looking after your own wellbeing, stress levels etc)

Do you feel that your ability to provide support for your family/child has changed? How has this come about, and what has changed?

What is your overall opinion about Parental Minds? What are the good bits and the bad bits?

Do you use other services or see any specialists to help support you outside of Parental Minds? Would you mind telling me what they are?

- How do they compare with Parental Minds (do they do the same thing or are there unique benefits to either?)

As researchers we're interested in collecting qualitative research like we're doing now in this interview, and quantitative data such as numbers and statistics. What should we be looking to measure in terms of numbers to show improvements due to parental minds? If we wanted to demonstrate the impact Parental Minds has had on you and other families, what do you think is the best way for us to show this? In other words, what should we be looking at to show improvements?

- (*hear their ideas first then suggest and get their thoughts on the following)*
- Some ideas might include caregiver mental health, child mental health and the number of times you need to access crisis services or other emergency services

What is the best way for us to ask you or other caregivers like you for this information?

- Eg. In person interviews, through WhatsApp/messaging, online surveys, being asked directly by researchers or by Parental Minds staff)

Is there anything else you'd like to say about Parental Minds that we haven't asked about?
